# Immune checkpoint inhibitor-associated interstitial nephritis: let’s move forward addressing the unknowns

**DOI:** 10.1093/ckj/sfag333

**Published:** 2026-09-24

**Authors:** Julie Belliere, Ben Sprangers

**Affiliations:** Department of Nephrology and Organ Transplantation, Referral Centre for Rare Kidney Diseases, French Intensive Care Renal Network, University Hospital of Toulouse, France; Toulouse University, Toulouse, France; INSERM U1297, Institute of Metabolic and Cardiovascular Diseases, Toulouse, France; Department of Nephrology, Ziekenhuis Oost-Limburg, Genk, Belgium; Faculty of Medicine and Life Sciences, Hasselt University, Diepenbeek, Belgium

**Keywords:** acute interstitial nephritis, immune checkpoint inhibitors, immunosuppression, kidney biopsy, onconephrology

## Abstract

Immune checkpoint inhibitors (ICIs) are now well recognized as a cause of acute interstitial nephritis (ICI-AIN), and their increasingly widespread use explains the growing incidence of reported cases. This clinicopathological entity is characterized by acute kidney injury with a tubulointerstitial pattern occurring in a patient exposed to an ICI, and histological confirmation is required to establish the diagnosis. Histopathological examination typically reveals a lymphocytic infiltrate, supporting the use of corticosteroid therapy for ∼12 weeks, which enables recovery of kidney function in >80% of cases. Although current recommendations are relatively well established, daily management remains challenging for nephrologists in a number of patients. In practice, nephrologists are not dealing with a single form of nephritis, but rather with several distinct entities, whose identification is progressively emerging through the literature, particularly with the description of different histopathological subtypes. We therefore sought to highlight specific patient populations for whom diagnostic, therapeutic, and prognostic management remains difficult despite the available literature.

## INTRODUCTION

Since the first descriptions of renal toxicity associated with the use of immune checkpoint inhibitors (ICIs) [1], the number of cases continues to rise steadily. A global burden of anticancer drug-induced acute kidney injury (AKI) and acute interstitial nephritis (AIN) has been detected, primarily due to ICI [2]. As indications for ICI in oncology are increasing, nephrologists are being consulted by oncologists more and more to assess the role of ICI in the pathogenesis of AKI and to determine the optimal management strategy. Two competing objectives must then be balanced: continuation of ICI for oncologic benefit, alongside preservation of kidney function, which ensures access to certain treatment lines and may influence overall prognosis [3]. Therefore, a dialogue begins between the oncologist and the nephrologist to decide on the management of patients who are highly heterogeneous, both in their oncologic history and in their renal presentation.

Today, in the setting of AKI occurring during ICI therapy, a kidney biopsy is recommended. If the biopsy reveals a lymphocytic infiltrate with tubulitis, a diagnosis of ICI-related AIN (ICI-AIN) is established, and the ICI is discontinued. The patient is started on corticosteroid therapy with a tapering course lasting <3 months. In >80% of cases, renal response is satisfactory [4], and the patient may resume immunotherapy 3 months after the renal adverse event, with a relative low risk of relapse [4] (“*End of story*”). For most patients and nephrologists, this nosological entity is therefore considered relatively straightforward. Guidelines from scientific societies are very clear regarding diagnostic procedures and have proposed an overall framework for corticosteroid treatment [5, 6].

However, individualized application of guidelines to a specific patient can still be challenging. We therefore sought to provide an overview of the clinical issues that remain unanswered, when diagnosing, predicting, and treating a patient with ICI-AIN. We will deliberately set aside the field of glomerular diseases which remain marginal (<10% of cases), electrolyte disorders, which are rarely problematic for oncologic management, and the issue of solid organ transplant recipients, for whom the pathophysiology is distinct.

## DIAGNOSIS

### Kidney biopsy management

#### Kidney biopsy relevance

Although ICI-AIN affects <5% of patients exposed to ICI [7, 8], its early identification has major implications, since its occurrence generally warrants interruption of oncologic treatment. Diagnosis usually relies on a classical approach requiring exclusion of obstructive and functional causes, and confirmation of a predominantly tubular or tubulointerstitial pattern of injury. The challenge is that many patients are simultaneously exposed to other potentially nephrotoxic agents, particularly platinum-based chemotherapy or pemetrexed, which may also impair kidney function. Distinguishing ICI-AIN from isolated acute tubular necrosis (ATN) without inflammatory lesions is therefore crucial [9].

Risk factors for ICI-AIN already identified in the literature include exposure to concomitant medications (e.g. proton pump inhibitors (PPIs), nonsteroidal anti-inflammatory drugs, antibiotics, fluindione), the presence of a genitourinary malignancy, and pre-existing chronic kidney disease [4, 10–12]. ICI combination also increases the risk, as does a prior history of or co-occurrence of a non-renal immune-related adverse event (irAE) [4]. The timing may also be suggestive as ICI-AKI typically occurs after ∼14 weeks of ICI exposure [13].

Regarding biological presentation, some markers are more frequently observed in patients with ICI-AIN, including elevated C-reactive protein (CRP) and leukocyturia. Peripheral eosinophilia has been reported in only a minority of patients with ICI-AIN, generally around 15%–20% in large cohorts [4]. Initial studies suggested that increased renal cortical FDG uptake on PET/CT might represent a non-invasive marker of ICI-AIN. However, more recent biopsy-validated data failed to demonstrate sufficient diagnostic accuracy to reliably distinguish ICI-AIN from other causes of AKI [14, 15].

Ultimately, at present, therapeutic decision-making remains difficult without kidney biopsy, which continues to represent the gold standard and is recommended by scientific societies [5, 6]. An empirical approach consisting of discontinuing the ICI and initiating corticosteroid therapy without histological confirmation may appear attractive because it avoids the risks associated with an invasive procedure. However, unnecessary exposure to corticosteroids—particularly in patients who actually have isolated ATN without inflammatory infiltrates—may result in significant morbidity and mortality. Not only may the patient be inappropriately deprived of immunotherapy, but they may also experience corticosteroid-related adverse effects, including reduced oncologic efficacy of immunotherapy, as well as classical complications such as steroid-induced diabetes, osteoporosis, and infections. Despite existing recommendations, the empirical practice of prescribing corticosteroids without performing a kidney biopsy is becoming increasingly common, sometimes even while continuing immunotherapy simultaneously, which represents both a pharmacological and immunological nonsense. One of the major challenges ahead is therefore to strengthen continuing education for nephrologists and oncologists in order to ensure consistent implementation of guidelines across healthcare territories, and thereby reduce inequalities in access to care for oncology patients (Fig. 1).

**Figure 1:**
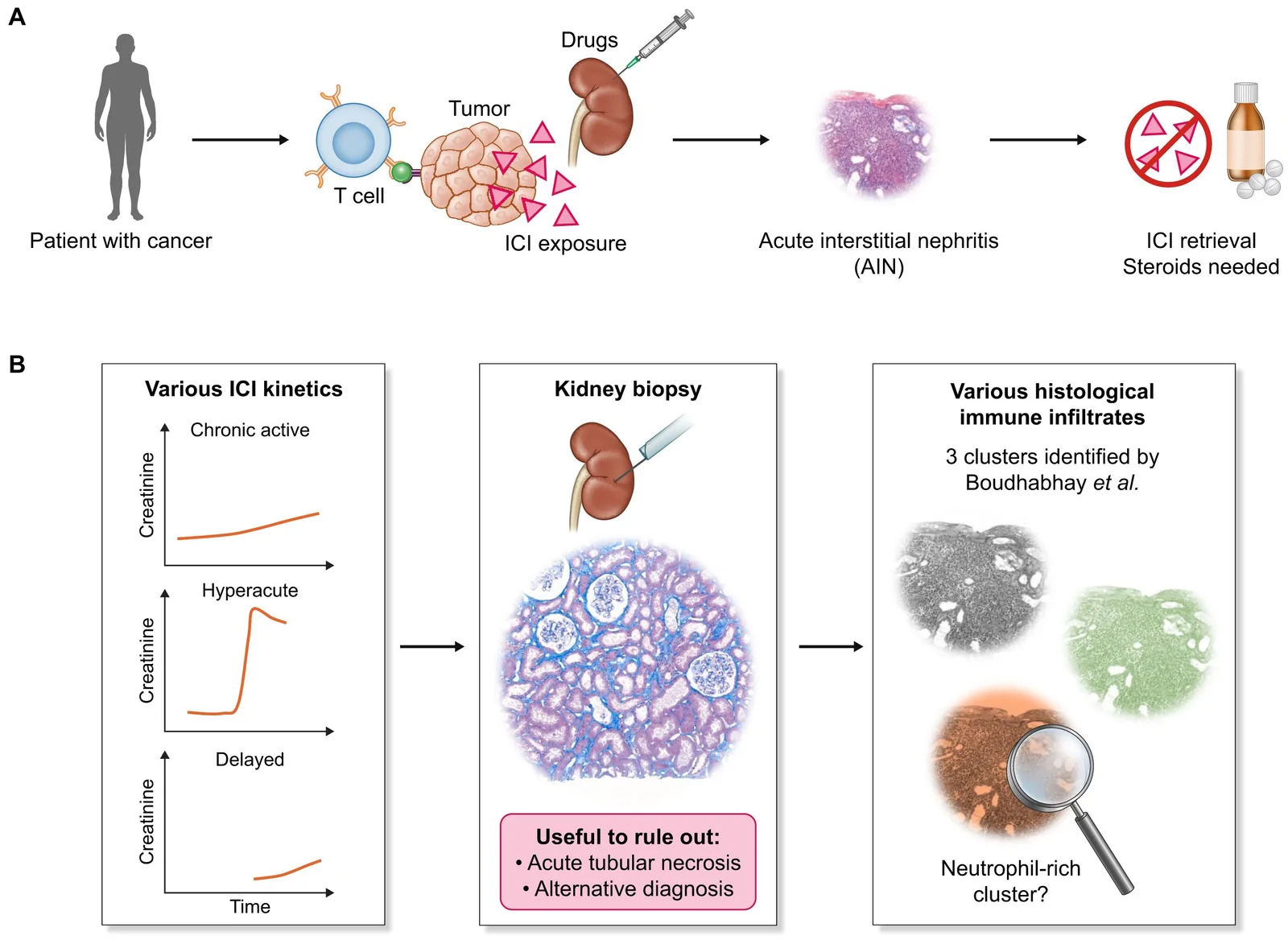
Current challenges in ICI-AIN identification in patients with acute kidney injury. Panel A: easy evolution of a patient with ICI-AIN suspicion, confirmation on biopsy, and standard of care combining ICI retrieval and steroids. Panel B: various clinical presentations with distinct creatinine kinetics, requiring kidney biopsy to rule out the major alternative diagnosis which is acute tubular necrosis (ATN). Once performed, kidney biopsy may predict renal outcome by identifying worse prognostic factors such as neutrophil-rich infiltrates (according to Boudhabhay *et al* [28]).

#### Performing kidney biopsy: a benefit–risk discussion

As depicted in Fig. 2, kidney biopsy should be considered the reference diagnostic procedure whenever it is technically feasible and clinically safe, particularly in patients with atypical presentations: cases of severe AKI during ICI treatment without another obvious explanation for the AKI, when multiple nephrotoxic drugs are involved, or when immunological abnormalities are present (e.g. monoclonal gammapathy, auto-antibodies suggestive for auto-immune disease like anti-SSA or -SSB for Sjögren syndrome), significant proteinuria or hematuria, or before initiating prolonged or second-line immunosuppressive therapy.

**Figure 2:**
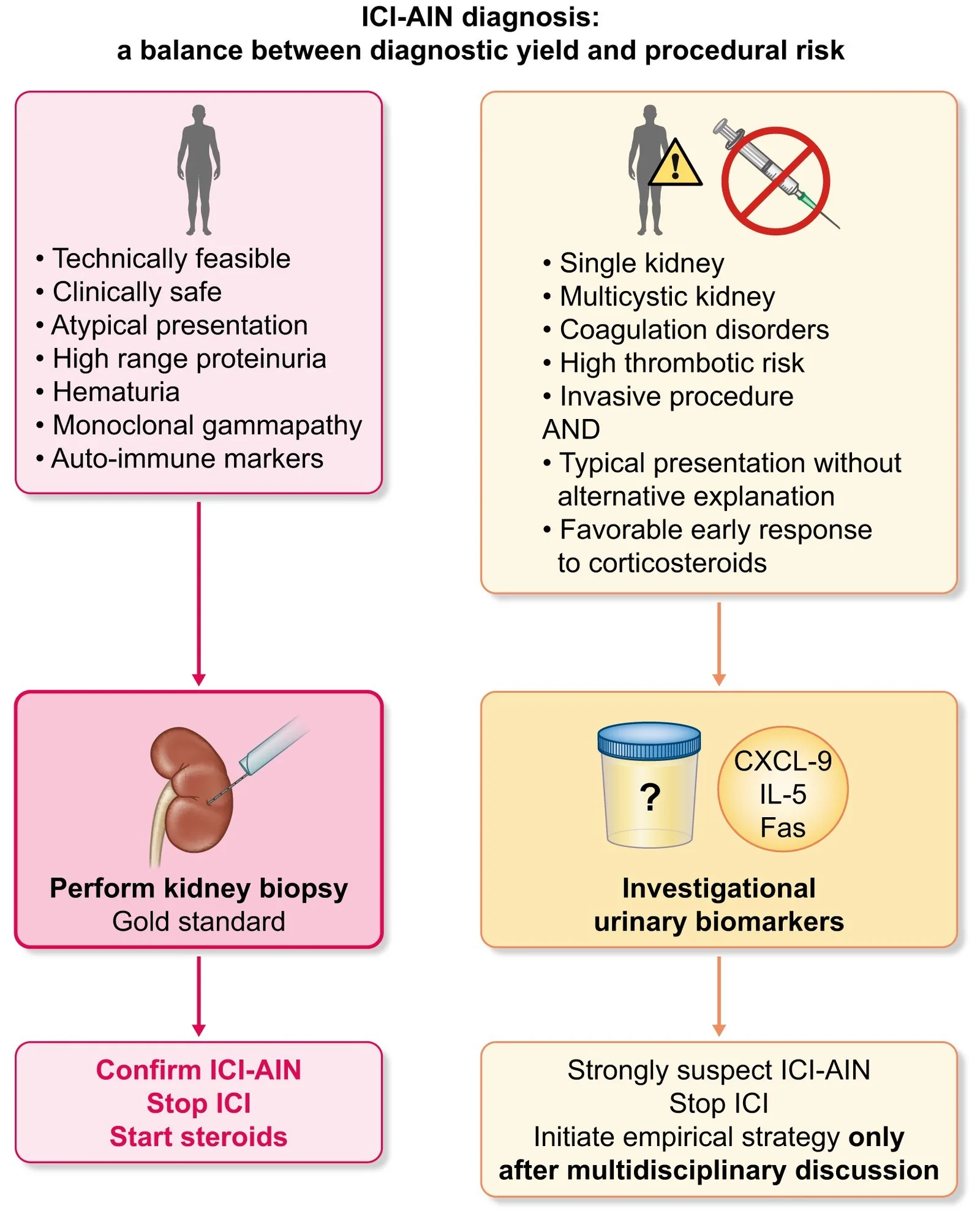
Suggested algorithm to diagnose ICI-AIN. Biomarkers such as Chemokine ligand 9 (CXCL-9) Interleukin 5 (IL-5) and Fas (FasR or CD95) are promising but remain investigational.

Conversely, we acknowledge that—unfortunately—many cancer patients have contraindications to perform a kidney biopsy, particularly those with a solitary kidney in the context of renal cancer, multicystic kidneys, or coagulation disorders. A history of thrombosis is also very common among patients referred for suspected AIN, including catheter-related thrombosis or recent deep vein thrombosis complicated by pulmonary embolism. Interrupting anticoagulation therapy in these patients is associated with a risk of recurrent thrombotic events, potentially severe, further increased by the bed rest required after kidney biopsy. Thus, in carefully selected patients with a typical clinical presentation of suspected ICI-AIN, no alternative explanation for AKI, contraindications to kidney biopsy, and a favorable early response to corticosteroids, a presumptive clinical diagnosis and empirical treatment may represent a reasonable strategy after multidisciplinary discussion. The decision to perform a kidney biopsy should always be individualized based on the balance between diagnostic yield and procedural risk.

#### Investigational use of urinary biomarkers

Urinary biomarkers have naturally emerged as promising candidates for distinguishing AIN from isolated ATN. Urine is an easily accessible and a non-invasive biological fluid, giving rise to the concept of the “liquid biopsy”. Several urinary biomarkers are already integrated into routine clinical practice for other kidney diseases, such as urinary CD163 in lupus nephritis or ANCA-associated vasculitis [16, 17]. Studies on biomarkers for interstitial nephritis had already been conducted before the advent of ICI, but the challenge of developing a non-invasive urinary score with sufficient diagnostic performance to replace kidney biopsy has become particularly critical. Table 1 summarizes the data on investigational urinary biomarkers for the diagnosis of ICI-AIN. Most of them deal with urinary chemokines, especially Interferon gamma-induced cytokines, namely CXCL9, CXCL10, and CXCL11, which play an important role in the antitumor immune response. Increased urinary levels of CXCL9 and CXCL10, both amplified by TNF-α, have already been described in AIN (not necessarily ICI-AIN).

**Table 1:** Investigational urinary biomarkers for the diagnosis of ICI-AIN.

Study (year)	Urinary biomarker	Total population (*n*)	AIN population (*n*)	Biopsy-proven AIN	Study design	ROC AUC	Diagnostic threshold	Interpretation of main conclusion
Moledina *et al*. [57]	TNF-α + IL-9	218	32	Yes	Prospective	0.84	Not reported	Demonstrates that urinary inflammatory cytokines can differentiate AIN from other causes of AKI with moderate-to-good accuracy
Moledina *et al*. [58]	TNF-α + IL-9	55	51	Yes	Retrospective	N/A	IL-9 > 0.66 ng/g	IL-9 levels were associated with lower 6 m-eGFR only in the subset of patients who did not receive corticosteroids
Singh *et al*. [59]	Urinary T cells clonotype analysis	5	5	4/5	Retrospective	N/A	N/A	Clonotype analysis of T cell receptor (TCR) sequences confirmed enrichment of kidney TCRs in urine
Isik *et al*. [60]	uRBP/Cr	50	37	38%	Retrospective	Median 1927 ug/g	Not reported	that serum CRP and uRBP/Cr may help to differentiate AKI due to ICI from other causes
Moledina *et al*. [22]	CXCL9	88	31 AIN	Yes	Prospective	0.77	>58.9 ng/g	Identifies CXCL9 as the most robust urinary biomarker for ICI-AIN
Gomez-Preciado *et al*.[62] CKJ 2024	Soluble PD-1 (sPD-1)	58	19	Partial	Retrospective independant cohort (*n*-30)	NR	usPD-1 < 129.3 pg/ml had a 71.43% sensitivity and 94.44% specificity to differentiate ATN from ICI-AIN	suggests a role of usPD-1 as non-invasive biomarker to differentiate ICI-AIN from ATN
Gupta *et al*. [23]	CXCL9	116	38	Partial	Prospective	0.84–0.90	269.5 ng/g	Validates CXCL9 as a strong and reproducible urinary biomarker for ICI-AIN
Long *et al*. [18]	IL-5 + Fas proteomic signature	49	22	Partial	Prospective	0.94	NULISA proteomic platform	Demonstrates that multiplex urinary proteomic signatures outperform single biomarkers for ICI-AIN diagnosis
Nava Chavez *et al*. [20]	CXCL9 vs TNF-α	∼92 + validation cohort	45	Partial	Prospective + validation	0.83 (CXCL9)	30.6 ng/g	Confirms superiority of CXCL9 over TNF-α and supports external validation of urinary chemokine signatures
Gomez Preciado *et al*. [19]	CXCL-5,9,10,11,CCL-2, CCL-3, CCL-5…	35 AIN 29 non ICI-AIN 26 ATN	35	Partial	Prospective	0.71 (CXCL-9)	20 pg/ml	Confirmation of CXCL-9 performances Longitudinal behavior of biomarkers

The area under theReceiver Operating Characteristic (ROC) curves (AUCs) reported in studies are generally around 0.8, with a maximum of 0.94 obtained for a proteomic signature combining IL-5 and Fas [18]. In a recent study by Gomez-Preciado *et al*. [19], in order to determine whether traditional biomarkers are useful by themselves or in combination with novel biomarkers, the authors first performed a multivariable model using the variables that met the inclusion criteria of *P* < 0.3 (creatinine, CRP, and number of leukocytes in urine) and also adjusted by sex and age. The clinical model yielded an AUC of 0.81. Afterward, they decided to add to the clinical model the combination of biomarkers usPD-1 and CXCL11 to see if the clinical model was improved by them. This combined model yielded the highest AUC of 0.91 [19], which is highly promising.

However, several important limitations have been raised regarding these approaches:

- Patient selection, which must be rigorous and include well-defined control groups. Patients with ICI-AKI due to ATN should be compared to ICI-AKI due to AIN. Included cases should rely only on histologically confirmed diagnoses. Infections of urinary tract should also be screened because inflammatory processes could bias biomarkers, especially chemokine levels.
- Pre-analytical sample processing, which represents a critical issue. Urine samples should ideally be collected before initiation of corticosteroid therapy, which was not the case in several studies, where up to 58% of patients had already received steroids depending on the cohort [20]. The stability of the studied molecules must also be characterized to ensure appropriate pre-analytical conditions, as it has been done for CXCL9 and CXCL10 [21]. Interpretation should consider the delay between urine collection and laboratory freezing. The centrifugation step, intended to separate the cellular pellet from urinary supernatant, should also be clearly described, as immune cells can be detected in urine, particularly in the setting of an inflammatory nephropathy such as AIN. Finally, concomitant urinary tract infection has not been precisely evaluated, although bacteriuria and urinary infections are relatively common in this patient population.
- Analytical technologies used for biomarker quantification. Several methods have been explored, including mass spectrometry, custom ELISA assays, ELLA technology, and Luminex platforms. However, only few studies have evaluated correlations between cytokine expression in kidney tissue and urinary concentrations [22].
- Interpretation of results and determination of diagnostic thresholds. At present, each study proposes its own cut-off value, but no consensual threshold has yet been formally validated. Thresholds may vary according to the clinical question being addressed. An optimal threshold for diagnosing interstitial nephritis in general may not necessarily be appropriate for ICI-AIN specifically, and some authors have suggested redefining cut-offs according to the clinical context. For example, Moledina *et al*. identified a threshold of 22.9 ng/g [3, 22], whereas Gupta *et al*. reported a threshold of 269.5 ng/g [23] for urinary CXCL-9.
- Normalization to urinary creatinine or proteinuria. Although it has been clearly established, particularly for soluble urinary CD163, that the degree of proteinuria can influence biomarker interpretation [16], normalization to proteinuria was not performed in most studies.

Despite encouraging preliminary results, no urinary biomarker is currently validated for routine clinical practice in patients with suspected ICI-AIN. Their use should therefore be considered investigational and restricted to research settings until prospective multicenter studies establish their diagnostic performance, reproducibility, and clinical utility.

### Kidney biopsy interpretation

#### Classification of distinct histologic subtypes

When a kidney biopsy is performed, it most often provides diagnostic confirmation, however, ICI-AIN should not be viewed as a uniform lymphocyte-driven entity, but rather as a spectrum of immune-defined subtypes characterized by distinct inflammatory programs and renal trajectories (Fig. 2). This heterogeneity may help explain the variability in outcome and corticosteroid responsiveness observed in clinical practice [24]. Several chronological presentations have been observed, ranging from acute and easily recognizable cases to more indolent forms, in which kidney biopsy reveals chronic active lesions. Hyperacute presentations have also been observed, particularly in patients exposed to triggering factors.

Although the principal histological feature remains a polymorphous lymphocytic infiltrate, its diagnostic and prognostic significance still requires clarification. It is acknowledged that increased T and B cells, Th1-CD8^+^ T cell axis accompanied by interferon-γ and TNF superfamily signatures are detected in ICI-AIN kidneys. Tertiary lymphoid structure gene signatures are also significantly increased in AIN cases and supported by histopathological identification [25]. A recent study suggests the use of a modified Banff classification to grade T infiltrates [26]. Systematic immune phenotyping is recommended to avoid certain diagnostic pitfalls, particularly low-grade B-cell hematologic malignancies [27]. The very recent work by Boudhabhay *et al*., based on multiplex immunofluorescence [28], used an unsupervised clustering approach and identified three immune phenotypes: a cluster with few mononuclear cells (Cluster 1), a cluster rich in mononuclear infiltrates (Cluster 2), and a neutrophil-rich cluster (Cluster 3), which was associated with greater systemic inflammation, reflected both by circulating markers such as CRP and by the neutrophil-to-lymphocyte ratio.

An upcoming study using spatial transcriptomic analyses further highlights the major role of interferon gamma-producing CD8^+^ T cells in ICI-AIN [29]. The authors describe distinct immunological niches, some enriched in plasma cells, others in CD8^+^ T cells, and others in myeloid cells. These analyses highlight the role of chemokines in the spatial organization of immune infiltrates in ICI-AIN, with CXCR3/CXCL9 interactions in areas of myeloid inflammation and CXCR5/CXCL13 interactions in tertiary lymphoid structures. Granulomatous forms have also been described, although metatranscriptomic analyses failed to detect infectious agents [28]. The clinical and prognostic significance of granulomas therefore remains to be determined.

Finally, interpretation of inflammatory infiltrates remains particularly challenging in chronic active, subtle, indolent, or late-diagnosed forms, in which extensive fibrosis itself may contribute to immune cell infiltration. In this context, additional immunological markers deserve further investigation, particularly PD1 or PD-L1 staining, which have been identified as potent discriminators [30–32].

To date, a precise classification of ICI-AIN is not yet available, limiting the feasibility of large-scale studies and introducing potential selection and interpretation biases across participating centers. A classification specifically adapted to the context of ICI-AIN should be developed and validated by scientific societies.

## TREATMENT

### Dose and duration

Once the histological diagnosis has been established, management consists of discontinuing the ICI and initiating systemic corticosteroid therapy. Current guidelines allow for relative flexibility in corticosteroid regimens in order to enable individualization of both dosage and treatment duration. Doses should not exceed 80 mg/day of prednisone or equivalent, and treatment durations beyond 8 weeks do not appear justified. Pulse corticosteroid therapy is reserved for severe forms with multiorgan involvement, while the intravenous route is preferred in patients with gastrointestinal involvement and/or concerns regarding treatment adherence [6]. At present, the choice of treatment duration is based neither on the type of immune checkpoint inhibitor nor on circulating drug levels. However, the half-life of immunotherapies varies considerably, with substantial heterogeneity across agents; avelumab has a relatively short half-life compared with pembrolizumab or atezolizumab (Table 2) [33]. It could be hypothesized that ICI with a longer half-life may necessitate a longer duration of corticosteroid therapy. However, no study to date has established a relationship between ICI half-life and renal response to corticosteroid treatment.

**Table 2:** Half-lives of the main ICI.

Class	Molecule	FDA Approval Date	Half-life (days)
Anti-CTLA-4	Ipilimumab	2011	15 days
Anti-PD-1	Nivolumab	2014	25 days
Anti-PD-1	Pembrolizumab	2014	22–27 days
Anti-PD-L1	Atezolizumab	2016	27 days
Anti-PD-L1	Durvalumab	2017	18–21 days
Anti-PD-L1	Avelumab	2017	4–6 days
Anti-PD-1	Cemiplimab	2018	19 days
Anti-PD-1	Dostarlimab	2021	∼25 days
Anti-CTLA-4	Tremelimumab	2022	20 days
Anti-LAG-3	Relatlimab	2022	∼9–18 days

To date, three studies have compared corticosteroid regimens. The first compared ∼10 patients treated for 3 weeks versus 6 weeks and did not demonstrate a significant difference [34]. The second compared 165 patients treated with corticosteroids, one third received a shorter duration of treatment (28 days or less) and two thirds received a longer duration (29–84 days). Patients in the shorter versus longer duration groups were similar with respect to baseline and ICI-AKI characteristics. Recurrence rate and renal outcome were similar in both groups. However, a large number of patients included in this study did not undergo a kidney biopsy, which can preclude general conclusion [35]. The third included 62 patients categorized into two groups receiving corticosteroid therapy for <12 weeks or >12 weeks. Prolonged corticosteroid treatment beyond 12 weeks was associated with a higher rate of complications [36]. Taken together, in the absence of randomized controlled clinical trials addressing corticosteroid dosing in ICI-AIN, the steroid regimen should be individualized based on the patient’s tolerance to corticosteroids and renal response, without lasting >12 weeks.

### Patients with problematic profile

Not only are the forms of nephritis heterogeneous, but the patient profiles themselves are also highly diverse, depending on the type of cancer and the treatments received. Corticosteroid therapy can rapidly become problematic in highly fragile patients. The following non-exhaustive list summarizes complex clinical situations that require multidisciplinary expert discussion, as strict application of current guidelines may be challenging for them (Fig. 3):

- Patients at high infectious risk who have already experienced complications under corticosteroid therapy, such as herpes zoster or *Pneumocystis jirovecii* infection. In this context, anti-infectious prophylaxis during corticosteroid treatment remains poorly standardized. Among the available alternatives, sulfamethoxazole-trimethoprim is not favored because of allergy risks. Pentamidine aerosols may be problematic because of respiratory adverse effects. Atovaquone or Dapsone remain options used in practice, although with limited evidence regarding either efficacy or tolerance. No dedicated study has specifically addressed this issue.
- Patients with a history of or active peptic ulcer disease. Exposure to PPIs triples the risk of AIN during immunotherapy [11, 37–40]. Maintaining PPIs is therefore potentially harmful once interstitial nephritis has developed. PPI-associated ICI-AKI appears to have a better prognosis than non-drug-related forms, suggesting that withdrawal of the triggering medication is beneficial. However, some patients may subsequently develop digestive complications related either to stress or to corticosteroid therapy itself, requiring reintroduction of PPIs. This raises the difficult question of balancing the renal contraindication to PPIs against the risk of gastrointestinal bleeding, which can sometimes be severe in patients who are frequently receiving therapeutic anticoagulation for thrombotic complications (as previously discussed in relation to biopsy-associated bleeding risk). When PPIs are contraindicated or considered inappropriate, H2 receptor antagonists may represent a reasonable alternative [41].
- Patients with a formal contraindication to corticosteroids. Some patients present with psychotic disorders or ophthalmologic complications for which corticosteroid therapy is formally contraindicated. In these cases, it remains unclear whether second-line treatments should be used upfront as first-line therapy.
- Patients developing AKI despite ongoing corticosteroid therapy, administered for extrarenal immune-related adverse events or brain metastases. Such patients should probably be considered primary steroid non-responders and evaluated early for second-line treatment.

**Figure 3:**
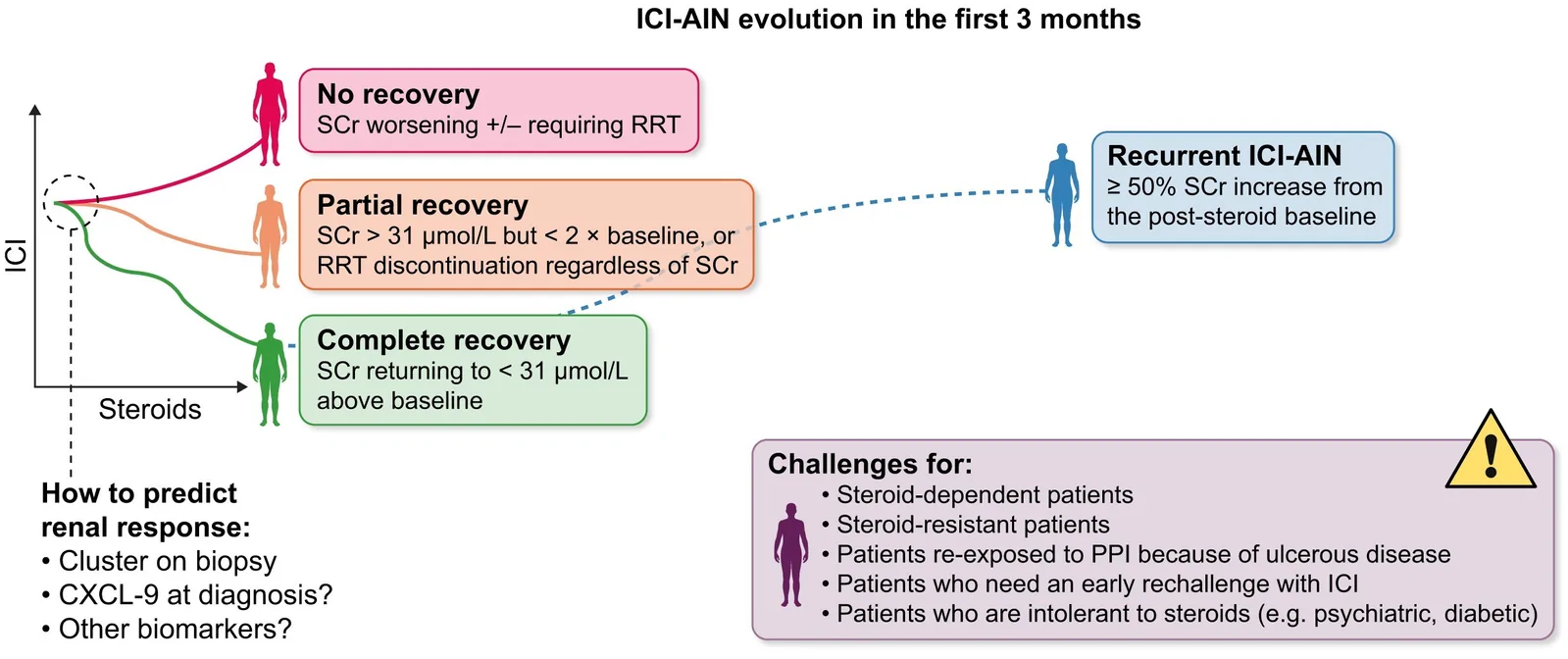
ICI-AIN evolution in the first 3 months. According to serum creatinine (SCr) variations, renal response will be classified as complete, partial, or no recovery. RRT: renal replacement therapy ; PPI: proton pump inhibitors. Special attention should be given to five patient’s profiles that are mentioned.

These situations illustrate that several patient profiles are poorly compatible with prolonged or high-dose corticosteroid exposure. This raises the question of second-line therapies, whose use could either spare corticosteroids or allow their discontinuation. According to current literature, several agents have already been explored.

### Which second-line immunosuppressive therapy should be selected?

Infliximab, an anti-TNF agent, is currently the molecule for which the greatest amount of evidence is available [42] (Table 3). Historically, development of TNF knockout mice proved that lack of TNF was protective against skin tumors [43]. TNF blockade enhanced antitumor immunity and overcame resistance to anti-PD-1 therapy [44]. Recently, *in vitro* models mimicking ICI-AIN demonstrated that selective TNF-α blockade prevents the development of nephritis. Following TNF-α inhibition, reductions in tissue interferon gamma as well as plasma cytokines including IL-6, MCP-1, and TNF-α were observed. These human translational models support the use of TNF blockade for its potential protective effects [45].

**Table 3:** Infliximab as second-line treatment for ICI-AIN.

Study (year)	Design	Population (*n*)	Biopsy-proven AIN	Infliximab dose	Indication	Outcome	Key conclusion
Lin *et al*. [46]	Retrospective case series	10	Yes	5 mg/kg IV, single or 1–2 doses	Steroid-refractory ICI-AIN	Partial/complete recovery in subset	First evidence supporting infliximab in steroid-refractory ICI-AIN
Barbir *et al*. [47]	Case series	3	Yes	5 mg/kg IV (repeat dose in some cases)	Steroid-dependent ICI-AIN	Renal improvement	Suggests steroid-sparing role of infliximab
Youssef *et al*. [42]	Retrospective cohort	55	Yes	5 mg/kg IV (1–2 doses depending on response)	Grade III–IV ICI-AIN	Faster recovery vs steroids alone	Suggests benefit of early infliximab addition

Infliximab use for ICI-AIN has been initially described in a cohort of 10 patients who relapsed within 4 weeks after corticosteroid discontinuation; among them, four achieved complete renal response, four partial response, and two showed no response [46]. More recently, a retrospective study conducted by Youssef *et al*. compared two groups of patients, one treated with corticosteroids alone and the other receiving corticosteroids plus infliximab [42]. Time to renal response was significantly shorter in patients exposed to infliximab within the first 30 days of corticosteroid therapy. The number of infliximab infusions ranged from one to six, with one-third of patients receiving a single dose. Infliximab was initiated within 5 days. Although patients treated with infliximab were older and had more severe disease, renal recovery occurred earlier. These retrospective data suggest that TNF blockade may represent a possible alternative to prolonged corticosteroid therapy [47], however the sample sizes in existing studies are insufficient to support this therapeutic regimen as a first-line approach. In terms of safety, neutropenia, infections, and autoantibody formation have not been prominently reported.

Many uncertainties nevertheless remain unresolved: (i) the clinical significance of the more rapid renal response induced by Infliximab which could result in a reduced delay before ICI rechallenge, (ii) the optimal steroid tapering scheme while using infliximab, and (iii) the correct endpoint to safely stop infliximab (renal response or CXCL-9 negativation). The impact of infliximab on tumor progression in patients with ICI-induced AIN also warrants careful consideration.

Aside from Infliximab, several immunosuppressants have been reported to be effective to resolve cortico-resistant or -dependent ICI-AIN [48]. Available data are summarized in Table 4 for mycophenolate [49, 50] and tofacitinib [51]. These strategies remain marginal and require larger cohort to be confirmed, even if there is currently an immunologic rationale to use them [52].

**Table 4:** Other second line treatments for ICI-AIN.

Drug	Reference	Study type	Clinical context	Indication	Renal outcome
Mycophenolate mofetil (MMF) 1 g twice daily	Faieta *et al*. [50, 61]	Case report	metastatic CCRCC nivolumab	Refractory ICI-AIN	A refractory grade 3 renal irAEs was effectively controlled with long-term use of mofetil mycophenolate with ongoing regression of metastases
Mycophenolate mofetil (MMF)	Moku *et al*. [49]	Case report	ICI therapy (solid tumor, unspecified ICI)	Steroid-refractory ICI-AIN	Improvement in renal function after MMF initiation
Mycophenolate mofetil (MMF)	Gupta *et al*., multicenter ICI-AKI cohort [10]	15 out of 22 patients receiving non steroid immunosuppression	various	MMF used in minority of steroid-refractory cases	Partial or complete recovery in most patients
Tofacitinib	Shivaraj *et al*. [51]	Case report	Metastatic lung cancer under pembrolizumab	Severe steroid-refractory ICI-AIN	Improvement in renal function and steroid tapering

## PROGNOSIS

### Renal response definition

The prognosis of ICI-AIN has already been well characterized by large epidemiological cohorts. The consensus definition of renal response to corticosteroid therapy is the following [4, 53], depicted in Fig. 3:

- Complete recovery was defined as serum creatinine (sCr) returning to <31 µmol/l (0.35 mg/dl) above baseline.
- Partial recovery was defined as sCr >31 µmol/l but < 2 × baseline, or renal replacement therapy (RRT) discontinuation regardless of sCr.
- No recovery was defined as dialysis-dependent AIN.
- Recurrent ICI-AIN was defined as a ≥ 50% sCr increase from the post-steroid baseline, attributed to ICI by the treating physician.

However, the relevance of this definition may be questioned in light of certain forms of nephritis that can be hyperacute and recover very rapidly, whereas others may follow a more subacute course in which stabilization alone already represents a therapeutic response. This raises the question whether new criteria for renal response to corticosteroids should be redefined, taking into account histological specificities and possibly urinary biomarkers. One corollary of this issue is the time point at which renal response to corticosteroid therapy should be assessed. As early as 15 days, renal improvement is expected to begin. In cases of lack of improvement or even worsening despite treatment, repeat biopsy is considered in most situations. However, the indications for repeat kidney biopsy have not yet been collectively standardized.

### Predicting renal response

#### With clinical parameters

The data available in the literature are conflicting regarding the association between the severity of renal injury and renal recovery. What is currently known is that early initiation of corticosteroid therapy (within 3 days) and the presence of a drug-related trigger (such as PPIs) are associated with a favorable prognosis [4]. However, nephritis may be very severe yet rapidly reversible, whereas forms with delayed diagnosis may already exhibit fibrosis; although initially less severe, these cases are often more difficult to treat.

#### With histology

The first study addressing this question highlighted the importance of interstitial fibrosis and tubular atrophy, which was the only significant parameter associated with lack of renal response at 3 months [54]. Among the histopathological prognostic factors, the most comprehensive study is that conducted by Boudhabhay *et al*. [28], which showed that neutrophilic infiltrates were predictive of poor renal response. The “neutrophil rich” cluster of patients had higher levels of peak sCr than the other two clusters and histologically more interstitial immune infiltrate, particularly with significantly higher inflammation in interstitial fibrosis, tubular atrophy, and more peritubular capillaritis. The presence of granular casts and neutrophilic tubulitis was associated with poor prognosis, with only 40% renal response at 1 year and more frequent relapses.

#### With urinary chemokines

Beyond renal histology, there is a need for biomarkers that allow earlier and more precise assessment of renal response than sCr alone. To date, only one study has described the evolution of urinary chemokines during corticosteroid treatment, showing a relatively rapid decline in CXCL9 and CXCL10 levels [19]. To assess whether these molecules were associated with kidney recovery from AKI, and accounting for the severity of the initial injury, the authors calculated a ratio of serum creatinine at diagnosis to that at 1-year follow-up. This ratio was higher in patients with elevated CRP and leukocyturia, reflecting significant positive correlations with these parameters. Regarding urine biomarkers, the creatinine ratio also showed positive correlations with the chemokines CXCL9 and CXCL10. This suggests that the greater the decrease in biomarker levels, the better the renal function at 1 year. However, interpreting the decline in CXCL9 levels is challenging, as this marker may reflect corticosteroid exposure alone rather than true regression of the pathogenic immune infiltrate. Additional studies are needed to address this question, as well as the intra-individual variability in CXCL9 measurements. Finally, assessing renal function 1 year after an episode of nephritis is challenging, as patients—often with metastatic disease—are likely to experience additional renal injuries related to their oncologic course (chemotherapies, targeted therapies, iodine products).

### Recurrence after rechallenge

One of the key issues is the need to resume ICI therapy despite the occurrence of ICI-AIN. Indeed, despite the development of an irAE, it may sometimes be crucial for the patient to restart ICI treatment because of its oncological benefit. In such cases, the question of rechallenge arises, together with the modalities of re-exposure. A recent meta-analysis compiled the available literature data on rechallenge: a total of eight articles involving 208 patients were included in the analysis of recurrent AKI after ICI rechallenge. The incidence of recurrent AKI following ICI-AKI was 18.0% [54]. Regardless of whether steroid was used upon rechallenge, there was no impact on the risk of recurrent AKI [55].

Thus, neither switching to another ICI nor resuming treatment under corticosteroid cover are currently recommended strategies for rechallenge. Although the evidence remains limited, most of these relapses appeared to be steroid-responsive [4]. The risk may be reduced by complete avoidance of the implicated agents (e.g. PPIs) and enhanced monitoring. The potential role of infliximab in preventing ICI-AIN relapse during ICI rechallenge remains to be clarified and warrants further investigation, as it could represent a valuable alternative to corticosteroids, which may adversely affect oncologic outcomes by impairing ICI efficacy.

Taken together, these data suggest that the management of ICI-AIN requires close monitoring to allow treatment adaptation according to the evolution of kidney function. A proposed management algorithm is presented in Fig. 4. Following the diagnosis of ICI-AIN and initiation of corticosteroid therapy, sCr should be monitored weekly. After 15 days, if sCr fails to improve or continues to worsen, several aspects should be reassessed. First, the diagnosis should be confirmed, and the kidney biopsy re-evaluated to identify the predominant immune infiltrate, particularly to detect a neutrophil-rich pattern. Adherence to corticosteroid therapy should also be assessed, together with the presence of multisystem immune-related toxicities, especially myocarditis. Concomitant medications that may contribute to kidney injury, such as PPIs or trimethoprim-sulfamethoxazole, should be carefully reviewed. Where available, measurement of CXCL-9 may provide additional diagnostic or prognostic information.

**Figure 4:**
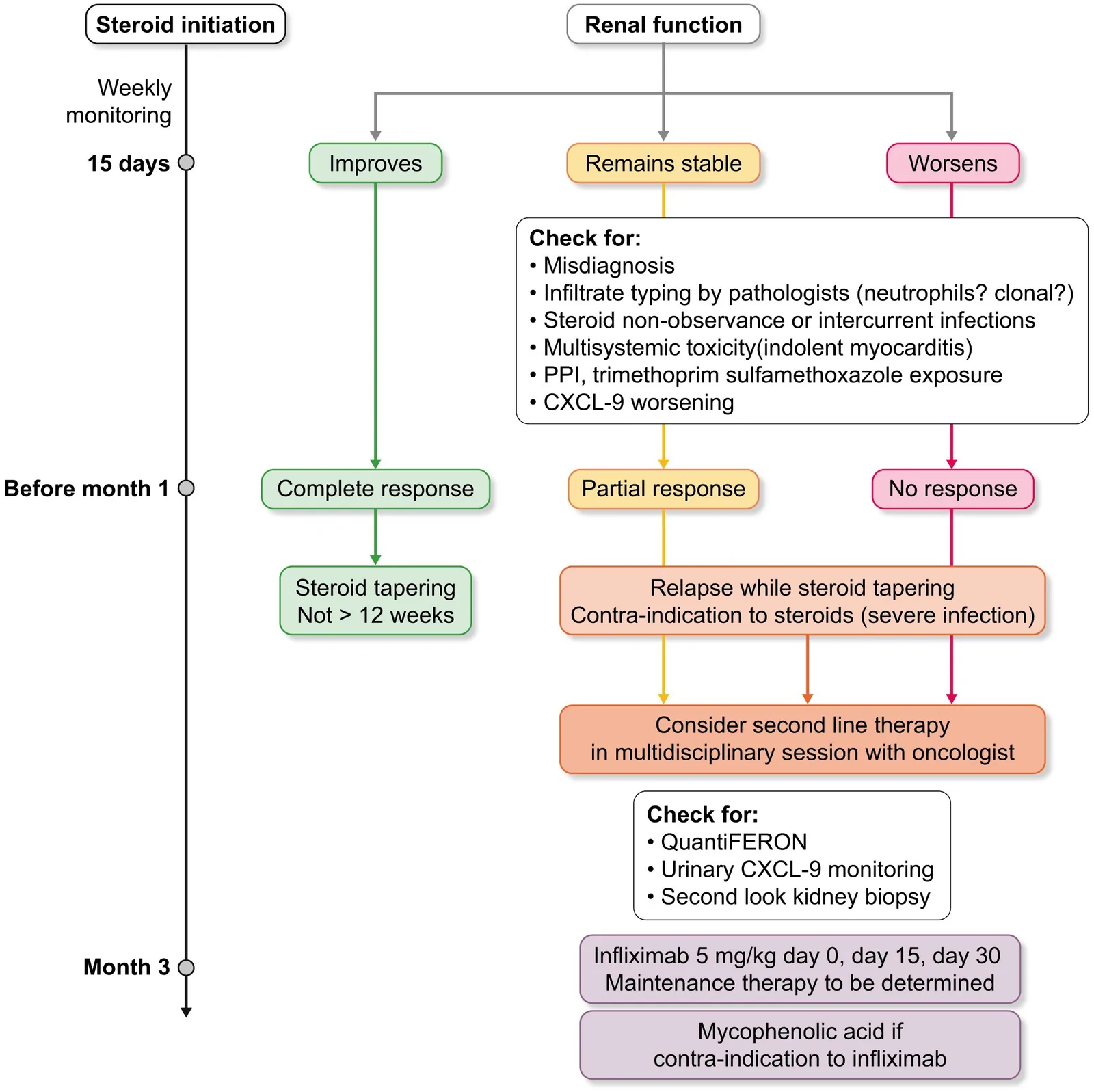
Suggested algorithm to manage ICI-AIN treatment accordingly to renal response. To date, there is no international guidelines on second line treatment because evidence-based literature is scarce.

After 1 month of corticosteroid therapy, the renal response should be assessed using the consensus criteria described above (see Fig. 3). In patients with a partial or absent renal response, those who relapse during corticosteroid tapering, or those with contraindications to prolonged corticosteroid therapy, second-line immunosuppressive treatment should be considered following multidisciplinary discussion involving nephrologists and oncologists. A repeat kidney biopsy should also be discussed. If infliximab is selected, latent tuberculosis screening (e.g. QuantiFERON testing) should be performed before treatment initiation. Mycophenolic acid may be considered when infliximab is contraindicated, although the supporting evidence remains very limited.

## BASIC RESEARCH

From a pathophysiological standpoint, the explanation for the onset of nephritis involves several successive insults, leading to a breakdown of tolerance toward renal tissue. However, basic science data are limited. Recent animal models have offered hope for a better understanding of the pathophysiological steps involved in the development of the disease. Available data are summarized in Table 5. A recent study evaluated the ability of a humanized immune system (HIS) tumor-bearing mouse model to investigate ICI-AKI [56]. Non-humanized Balb/c Rag2^−/−^ Il2rg^−/−^ Sirpa NOD (BRG) and humanized mice (HIS-BRG) were implanted with human breast cancer cells and treated with either nivolumab or a combination of nivolumab and ipilimumab. Histopathological analysis revealed that HIS-BRGS mice treated with ICI exhibited significant interstitial nephritis and vasculitis/periarteritis, consistent with kidney phenotypes observed in patients. These results suggest that mice can effectively recapitulate features of ICI-AIN observed in patients and can be used to study mechanisms and prevention strategies to limit irAEs. It is still not clearly understood why and how nephritis-inducing drugs are recognized by the immune system, nor what the shared epitopes are between tumor and renal epithelial cells. These results suggest that HIS-BRGS mice can effectively recapitulate features of ICI-induced kidney injury observed in patients and can be used to study mechanisms and prevention strategies to limit irAEs.

**Table 5:** Available murine models for ICI-AIN.

Reference	Mouse model	ICIs studied	Renal injury	Key findings
TNF-α blockade mitigates immune checkpoint-related nephritis in a humanized mouse model (Cuenca Narvaez *et al*.) [45]	Humanized PD-1/PD-L1 chimeric mice	Anti–PD-1/anti–PD-L1 ± pro-inflammatory cytokines	AIN	Model recapitulates human ICI-AIN; TNF-α blockade is protective
Pathological findings of immunotherapy-induced nephrotoxicity in a humanized immune system mouse model (Asby *et al*.) [56]	HIS-BRGS humanized mice with tumors	Nivolumab ± ipilimumab	Interstitial nephritis + vasculitis/periarteritis	Anti–PD-1 + anti–CTLA-4 worsens renal lesions
Immune checkpoint inhibitors induce acute interstitial nephritis in mice with increased urinary MCP1 and PD-1 glomerular expression (Martinez Valenzuela *et al*.) [61]	Experimental murine model of ICI-induced AIN	PD-1/CTLA-4 ± platinum salts	AIN	↑ urinary MCP-1 and ↑ glomerular PD-1 expression

## CONCLUSION

Although numerous studies have helped establish a framework for the diagnosis and management of ICI-AIN, many questions remain unresolved. To maintain the overarching goals of quality of life and patient safety, the nephrology community must continue to prioritize ICI-AIN as a key area of research focus. A composite score integrating clinical parameters and urinary biomarkers could be highly valuable for diagnosing ICI-AIN when kidney biopsy is not feasible. Furthermore, a better understanding of prognostic factors may help tailor immunosuppressive strategies and optimize the preservation of kidney function, which remains a major priority in many oncologic settings.

## Data Availability

No new data were generated or analyzed in support of this research.

## References

[1] Izzedine H, Gueutin V, Gharbi C et al. Kidney injuries related to ipilimumab. Invest New Drugs. 2014;32:769–73. 10.1007/s10637-014-0092-7 24687600

[2] Yoon S-Y, Lee S, Lee K et al. Global burden of anticancer drug-induced acute kidney injury and tubulointerstitial nephritis from 1967 to 2023. Sci Rep. 2024;14:16124. 10.1038/s41598-024-67020-x PMC11245615 38997405

[3] Péron J, Neven A, Collette L et al. Impact of acute kidney injury on anticancer treatment dosage and long-term outcomes: a pooled analysis of European Organisation for Research and Treatment of Cancer trials. Nephrol Dial Transplant. 2021;36:1263–71. 10.1093/ndt/gfaa049 32337562

[4] Cortazar FB, Kibbelaar ZA, Glezerman IG et al. Clinical features and outcomes of immune checkpoint inhibitor-associated AKI: a multicenter study. J Am Soc Nephrol 2020;31:435–46. 10.1681/ASN.2019070676 PMC7003302 31896554

[5] Herrmann SM, Abudayyeh A, Gupta S et al. Diagnosis and management of immune checkpoint inhibitor-associated nephrotoxicity: a position statement from the American Society of Onco-nephrology. Kidney Int. 2025;107:21–32. 10.1016/j.kint.2024.09.017 39455026

[6] Gueutin V, Dalle S, Isnard-Bagnis C et al. Acute kidney injury in cancer patients receiving immune checkpoint inhibitor therapy-shared guidelines of FITC/SFNDT. Bull Cancer. 2025;112:225–35. 10.1016/j.bulcan.2024.11.001 39643454

[7] Seethapathy H, Herrmann SM, Sise ME. Immune checkpoint inhibitors and kidney toxicity: advances in diagnosis and management. Kidney Med. 2021;3:1074–81. 10.1016/j.xkme.2021.08.008 PMC8664750 34939017

[8] Gougis P, Jochum F, Abbar B et al. Clinical spectrum and evolution of immune-checkpoint inhibitors toxicities over a decade-a worldwide perspective. EClinicalMedicine. 2024;70:102536. 10.1016/j.eclinm.2024.102536 PMC10981010 38560659

[9] Moledina DG, Parikh CR. Differentiating acute interstitial nephritis from acute tubular injury: a challenge for clinicians. Nephron. 2019;143:211–6. 10.1159/000501207 PMC6821567 31203275

[10] Gupta S, Short SAP, Sise ME et al. Acute kidney injury in patients treated with immune checkpoint inhibitors. J Immunother Cancer. 2021;9:e003467. 10.1136/jitc-2021-003467 PMC8496384 34625513

[11] Miao J, Herrmann SM. Immune checkpoint inhibitors and their interaction with proton pump inhibitors–related interstitial nephritis. Clin Kidney J. 2023;16:1834–44. 10.1093/ckj/sfad109 PMC10616479 37915905

[12] Gérard AO, Barbosa S, Parassol N et al. Risk factors associated with immune checkpoint inhibitor-induced acute kidney injury compared with other immune-related adverse events: a case-control study. Clin Kidney J. 2022;15:1881–7. 10.1093/ckj/sfac109 PMC9494514 36158153

[13] Tawhari I, Fenton SE, Sosman JA et al. Hyperacute Onset of Immune Checkpoint Inhibitor–Related Acute Interstitial Nephritis. Kidney Int Rep. 2020;5:2084–8. 10.1016/j.ekir.2020.08.002 PMC7609883 33163728

[14] Gupta S, Green-Lingren O, Bhimaniya S et al. F18-FDG PET imaging as a diagnostic tool for immune checkpoint inhibitor-associated acute kidney injury. J Clin Invest. 2024;134:102536. 10.1172/JCI182275 PMC11405038 39115940

[15] Lyrio RMDa, Narvaez VDC, Chavez CCN et al. F18-FDG PET-CT in immune checkpoint inhibitor-associated acute interstitial nephritis: what is the diagnostic value?. Clin Kidney J. 2026;19:sfag042. 10.1093/ckj/sfag042 PMC13006872 41877892

[16] Mejia-Vilet JM, Zhang XL, Cruz C et al. Urinary soluble CD163: a novel noninvasive biomarker of activity for lupus nephritis. J Am Soc Nephrol 2020;31:1335–47. 10.1681/ASN.2019121285 PMC7269356 32300067

[17] Moran SM, Scott J, Clarkson MR et al. The Clinical Application of Urine Soluble CD163 in ANCA-Associated Vasculitis. J Am Soc Nephrol 2021;32:2920–32. 10.1681/ASN.2021030382 PMC8806104 34518279

[18] Long JP, Singh S, Dong Y et al. Urine proteomics defines an immune checkpoint-associated nephritis signature. J Immunother Cancer. 2025;13:e010680. 10.1136/jitc-2024-010680 PMC11784134 39863302

[19] Gomez-Preciado F, Martinez-Valenzuela L, Anton-Pampols P et al. Urinary Chemokines in the Diagnosis and Monitoring of Immune Checkpoint Inhibitor-Associated Nephritis. Int J Mol Sci. 2026;27:1240. 10.3390/ijms27031240 PMC12898666 41683669

[20] Chavez CCN, Bermejo S, Narvaez VC et al. Urinary CXCL-9 outperforms TNF-α for non-invasive diagnosis of immune checkpoint inhibitor-associated acute interstitial nephritis. Nephrol Dial Transplant. 2026;41:1457–66. 10.1093/ndt/gfag016 41642247

[21] Tinel C, Sauvaget V, Aouni L et al. Transforming kidney transplant monitoring with urine CXCL9 and CXCL10: practical clinical implementation. Sci Rep. 2024;14:20357-. 10.1038/s41598-024-70390-x PMC11369285 39223175

[22] Moledina DG, Obeid W, Smith RN et al. Identification and validation of urinary CXCL9 as a biomarker for diagnosis of acute interstitial nephritis. J Clin Invest. 2023;133:e168950. 10.1172/JCI168950 PMC10313360 37395276

[23] Gupta S, Mistry K, Alikhan FM et al. Urinary C-X-C-motif ligand 9 (CXCL9) in immune checkpoint inhibitor–associated acute interstitial nephritis. Kidney Int. 2025;108:491–6. 10.1016/j.kint.2025.05.029 PMC12341002 40578686

[24] Boudhabhay I, Isnard P. Response to the letter to the editor entitled “Beyond lymphocytic inflammation: immune architecture as a determinant of renal outcomes.”. Kidney Int Rep. 2026;11:106385. 10.1016/j.ekir.2026.106385 PMC13058976 41958900

[25] Singh S, Long JP, Tchakarov A et al. Tertiary lymphoid structure signatures are associated with immune checkpoint inhibitor related acute interstitial nephritis. JCI Insight. 2022;36472921. 10.1172/JCI.INSIGHT.165108 36472921

[26] Al-Othman YA, Metcalf BD, Kroneman O et al. Grading T lymphocyte-mediated acute interstitial nephritis following checkpoint inhibitor therapy. Ann Clin Lab Sci. 2025;55:172–8. 40340877

[27] Benyahia R, Roques O, Ysebaert L et al. Pivotal role for typing of renal infiltrates: kidney localisation of a lymphoma presenting as pembrolizumab-associated nephritis. A lesson for the clinical nephrologist. J Nephrol. 2025;38:1487–91. 10.1007/s40620-025-02246-0 PMC12289724 40354006

[28] Boudhabhay I, Lazareth H, Oniszczuk J et al. Immune profiling identifies high-risk neutrophil-rich subtype in checkpoint inhibitor nephritis. Kidney Int Rep. 2026;11:103766. 10.1016/j.ekir.2025.103766 PMC12918167 41727781

[29] Qin Q, Ostendorf L, Wells SL et al. Spatial transcriptomics identify T cell-driven mechanisms of kidney damage in immune checkpoint inhibitor-associated acute interstitial nephritis. Kidney Int Rep. 2026;11:107007. 10.1016/j.ekir.2026.107007, PMC13583950 42756960

[30] Mancianti N, Tripodi SA, Pascucci A et al. Immunohistochemical evaluation of renal biopsy with anti-PD1 and p53 to solve the dilemma between platinum- and pembrolizumab-induced AKI: case report and review. J Clin Med. 2024;13:1828. 10.3390/jcm13071828 PMC11012688 38610593

[31] Casals J, Acosta Y, Caballero G et al. Differentiating acute interstitial nephritis from immune checkpoint inhibitors from other causes. Kidney Int Rep. 2023;8:672–5. 10.1016/j.ekir.2022.12.017 PMC10014382 36938095

[32] Hakroush S, Kopp SB, Tampe D et al. Variable expression of programmed cell death protein 1-ligand 1 in kidneys independent of immune checkpoint inhibition. Front Immunol. 2021;11:624547. 10.3389/fimmu.2020.624547 PMC7858644 33552089

[33] Centanni M, Moes DJAR, Trocóniz IF et al. Clinical pharmacokinetics and pharmacodynamics of immune checkpoint inhibitors. Clin Pharmacokinet. 2019;58:835–57. 10.1007/s40262-019-00748-2 PMC6584248 30815848

[34] Lee MD, Seethapathy H, Strohbehn IA et al. Rapid corticosteroid taper versus standard of care for immune checkpoint inhibitor induced nephritis: a single-center retrospective cohort study. J Immunother Cancer. 2021;9:e002292. 10.1136/jitc-2020-002292 PMC8051410 33849926

[35] Gupta S, Garcia-Carro C, Prosek JM et al. Shorter versus longer corticosteroid duration and recurrent immune checkpoint inhibitor-associated AKI. J Immunother Cancer. 2022;10:e005646. 10.1136/jitc-2022-005646 PMC9511654 36137651

[36] Auñón P, Lopes V, Minguez I et al. Short versus long corticosteroid therapy for immune checkpoint inhibitors–induced acute tubulointerstitial nephritis. Clin Kidney J. 2026;19:sfag076. 10.1093/ckj/sfag076 PMC13078163 41987871

[37] Tan HZ, Sprangers B. Proton pump inhibitors and adverse kidney outcomes during immune checkpoint blockade: time to sound the alarm?. Clin Kidney J. 2023;16:1709–13. 10.1093/ckj/sfad205 PMC10616439 37915924

[38] Seethapathy H, Zhao S, Chute DF et al. The incidence, causes, and risk factors of acute kidney injury in patients receiving immune checkpoint inhibitors. Clin J Am Soc Nephrol. 2019;14:1692–700. 10.2215/CJN.00990119 PMC6895474 31672794

[39] Cortazar FB, Marrone KA, Troxell ML et al. Clinicopathological features of acute kidney injury associated with immune checkpoint inhibitors. Kidney Int. 2016;90:638–47. 10.1016/j.kint.2016.04.008 PMC4983464 27282937

[40] Gérard AO, Andreani M, Fresse A et al. Immune checkpoint inhibitors-induced nephropathy: a French national survey. Cancer Immunol Immunother. 2021;70:3357–64. 10.1007/s00262-021-02983-8 PMC10991556 34155532

[41] Vakil N. Peptic ulcer disease: a review. JAMA. 2024;332:1832–42. 10.1001/jama.2024.19094 39466269

[42] Youssef N, Habeeb M, Chen H et al. Infliximab for grade III or IV immune checkpoint inhibitor nephritis clinical and translational evidence. Kidney Int Rep. 2026;11:32–42. 10.1016/j.ekir.2025.10.018 PMC12799554 41541786

[43] Moore RJ, Owens DM, Stamp G et al. Mice deficient in tumor necrosis factor-alpha are resistant to skin carcinogenesis. Nat Med. 1999;5:828–31. 10.1038/10552 10395330

[44] Bertrand F, Montfort A, Marcheteau E et al. TNFα blockade overcomes resistance to anti-PD-1 in experimental melanoma. Nat Commun. 2017;8:2256-. 10.1038/s41467-017-02358-7 PMC5741628 29273790

[45] Cuenca Narvaez VD, Chavez CN, Refai OA et al. TNF-α blockade mitigates immune checkpoint-related nephritis in a humanized mouse model. JCI Insight. 2026;11:e199694. 10.1172/jci.insight.199694 PMC13135400 41785046

[46] Lin JS, Mamlouk O, Selamet U et al. Infliximab for the treatment of patients with checkpoint inhibitor–associated acute tubular interstitial nephritis. Oncoimmunology. 2021;10:1–11. 10.1080/2162402X.2021.1877415 PMC7872057 33643693

[47] Barbir E-B, Mohan A, Koirala P et al. Biomarker-guided infliximab therapy for immune checkpoint inhibitor-induced acute interstitial nephritis. Nephrol Dial Transplant. 2025:40:602–604. 10.1093/ndt/gfae257 39516049

[48] Esposito P, Bottini A, Lecini E et al. Biopsy-proven acute tubulointerstitial nephritis in patients treated with immune checkpoint inhibitors: a pooled analysis of case reports. Front Oncol. 2023;13:1221135. 10.3389/fonc.2023.1221135 PMC10627243 37936605

[49] Moku P, Bakow B, Muthiah A et al. Steroid refractory immune checkpoint induced acute interstitial nephritis salvaged by mycophenolate mofetil. Brown J Hosp Med. 2023;2:74097. 10.56305/001c.74097 PMC11878859 40046150

[50] Faieta A, Dancik T. Case report refractory acute interstitial nephritis in the setting of Nivolumab therapy. Case Rep Nephrol. 2021;6640154. 10.1155/2021/6640154 PMC7939740 33728076

[51] Shivaraj K, Tchakarov A, Dong Y et al. Tofacitinib for the treatment of refractory immune checkpoint inhibitor-associated immune nephritis. Clin Kidney J. 2024;17:sfae127. 10.1093/ckj/sfae127 PMC11129582 38803394

[52] Zheng M-M, Li J-Y, Guo H-J et al. IMPDH inhibitors upregulate PD-L1 in cancer cells without impairing immune checkpoint inhibitor efficacy. Acta Pharmacol Sin. 2025;46:1058–67. 10.1038/s41401-024-01411-8 PMC11950507 39592732

[53] Yu Y, Biros E, Chang X et al. A systematic review of treatment for acute interstitial nephritis. Kidney Int Rep. 2025;10:2575–84. 10.1016/j.ekir.2025.05.009 PMC12347843 40814598

[54] Abudayyeh A, Suo L, Lin H et al. Pathologic predictors of response to treatment of immune checkpoint inhibitor–induced kidney injury. Cancers. 2022;14:5267. 10.3390/cancers14215267 PMC9656112 36358686

[55] Ho C-W, Kang N-W, Yeh T-H et al. Immune checkpoint inhibitors-associated acute kidney injury: a systematic review and meta-analysis of incidence, kidney recovery, and recurrent risk. Cancer Immunol Immunother. 2025;74:324. 10.1007/s00262-025-04147-4 PMC12484524 41026215

[56] Asby SC, Thompson LE, Goedken M et al. Pathological findings of immunotherapy-induced nephrotoxicity in a humanized immune system mouse model. Kidney Int. 2025;107:930–4. 10.1016/j.kint.2025.01.021 PMC12121629 39922378

[57] Moledina DG, Wilson FP, Pober JS et al. Urine TNF-α and IL-9 for clinical diagnosis of acute interstitial nephritis. JCI Insight. 2019;4:e127456. 10.1172/jci.insight.127456 PMC6542610 31092735

[58] Moledina DG, Wilson FP, Kukova L et al. Urine interleukin-9 and tumor necrosis factor-α for prognosis of human acute interstitial nephritis. Nephrol Dial Transplant. 2021;36:1851–8. 10.1093/ndt/gfaa169 PMC8476079 33125471

[59] Singh S, Clemente LC, Parra ER et al. Urinary T cells are detected in patients with immune checkpoint inhibitor-associated immune nephritis that are clonotypically identical to kidney T cell infiltrates. Oncoimmunology. 2022;11:2124678. 10.1080/2162402X.2022.2124678 PMC9519023 36185804

[60] Isik B, Alexander MP, Manohar S et al. Biomarkers, clinical features, and rechallenge for immune checkpoint inhibitor renal immune-related adverse events. Kidney Int Rep. 2021;6:1022–31. 10.1016/j.ekir.2021.01.013 PMC8071627 33912752

[61] Martinez Valenzuela L, Gómez-Preciado F, Guiteras J et al. Immune checkpoint inhibitors induce acute interstitial nephritis in mice with increased urinary MCP1 and PD-1 glomerular expression. J Transl Med. 2024;22:421. 10.1186/s12967-024-05177-9 PMC11069287 38702780

[62] Gomez-Preciado F, Martinez-Valenzuela L, Anton-Pampols P et al. Urinary soluble PD-1 as a biomarker of checkpoint inhibitor-induced acute tubulointerstitial nephritis. Clin Kidney J. 2024;17. 10.1093/ckj/sfae200 PMC11316395 39131079

